# Application of Lysine-specific Labeling to Detect Transient Interactions Present During Human Lysozyme Amyloid Fibril Formation

**DOI:** 10.1038/s41598-017-14739-5

**Published:** 2017-11-03

**Authors:** Minkoo Ahn, Christopher A. Waudby, Ana Bernardo-Gancedo, Erwin De Genst, Anne Dhulesia, Xavier Salvatella, John Christodoulou, Christopher M. Dobson, Janet R. Kumita

**Affiliations:** 1 Department of Chemistry, University of Cambridge, Lensfield Road, Cambridge, CB2 1EW UK; 2 Institute of Structural and Molecular Biology, University College London and Birkbeck College, Gower Street, London, WC1E 6BT UK; 3 ICREA and Institute for Research in Biomedicine (IRB Barcelona), The Barcelona Institute of Science and Technology, Baldiri Reixac 10, 08028 Barcelona, Spain

## Abstract

Populating transient and partially unfolded species is a crucial step in the formation and accumulation of amyloid fibrils formed from pathogenic variants of human lysozyme linked with a rare but fatal hereditary systemic amyloidosis. The partially unfolded species possess an unstructured β-domain and C-helix with the rest of the α-domain remaining native-like. Here we use paramagnetic relaxation enhancement (PRE) measured by NMR spectroscopy to study the transient intermolecular interactions between such intermediate species. Nitroxide spin labels, introduced specifically at three individual lysine residues, generate distinct PRE profiles, indicating the presence of intermolecular interactions between residues within the unfolded β-domain. This study describes the applicability to PRE NMR measurements of selective lysine labeling, at different sites within a protein, as an alternative to the introduction of spin labels via engineered cysteine residues. These results reveal the importance of the β-sheet region of lysozyme for initiating self-assembly into amyloid fibrils.

## Introduction

Globular proteins with cooperative and persistent folds under native conditions can form amyloid fibrils and cause protein deposition diseases as a result of global or partial unfolding of the structure^1,2^. Human lysozyme is a well-known glycosidase containing 130 amino acid residues^3^. In 1993, two mutational variants of human lysozyme were directly associated with a rare form of systemic amyloidosis^4^. Since then it has been found that a reduction in native state stability and global cooperativity of the pathogenic variants give rise to the increased population of the transient intermediate species which initiates protein aggregation into amyloid fibrils^5–7^. Under destabilizing conditions (high temperatures and low pH) we reported that the intermediate species appear to be an ensemble of interconverting conformers with varying degrees of denaturation^8^, and that the rate of seeded fibril formation by human lysozyme is directly proportional to the population of this state^9^. However, despite the importance of the transient intermediate species in fibril formation, it is highly challenging to investigate the initial events of the aggregation process due to the heterogeneous nature and low population of the intermediate species, making them recalcitrant to most biophysical techniques.

NMR spectroscopy has proved to be one of the most powerful techniques for investigating the structure of transient protein molecules present at low population^10,11^. A number of NMR methods with the ability to monitor such states have been developed, but two relaxation based methods stand out: paramagnetic relaxation enhancement (PRE) and relaxation dispersion (RD) NMR^12,13^. Whilst RD provides kinetic (exchange rates) and thermodynamic (population) information on systems that undergo chemical exchange on millisecond to microsecond timescales, PRE provides structural information through the measurements of distances between a paramagnetic center and the NMR active nuclei of interest. The last decade has seen significant advances in the application of PRE to a variety of biological systems^12^ including visualization of ultra-weak protein self-association^14^, and use of PRE to determine intermolecular protein interactions contributing to amyloid fibril formation^15,16^.

To investigate the intermolecular interactions between transiently populated human lysozyme molecules by PREs, we first need to incorporate appropriate probes. With the exception of metalloproteins that contain intrinsic paramagnetic groups, most proteins must be labeled with an extrinsic paramagnetic group in order to conduct PRE measurements and a range of spin labeling techniques have been applied to PRE measurements of proteins^12,17^. Introduction of paramagnetic groups into proteins includes the use of nitroxide spin labels (MTSL^18^, PROXYL^19^) and metal chelators (EDTA^20^, ATCUN^21^). Most commonly, MTSL is introduced via a thiol group, as it is often relatively straightforward to introduce a single cysteine residue into the protein of interest using site-directed mutagenesis. In the case of human lysozyme, however, this strategy is not practical due to the presence of eight cysteine residues which generate four disulfide bonds that are crucial for the native-state structural integrity^3,22^. Although in WT lysozyme, a spin label has previously been incorporated via its single histidine residue, the information obtained remains limited without further labeling sites^23,24^.

**Figure 1:**
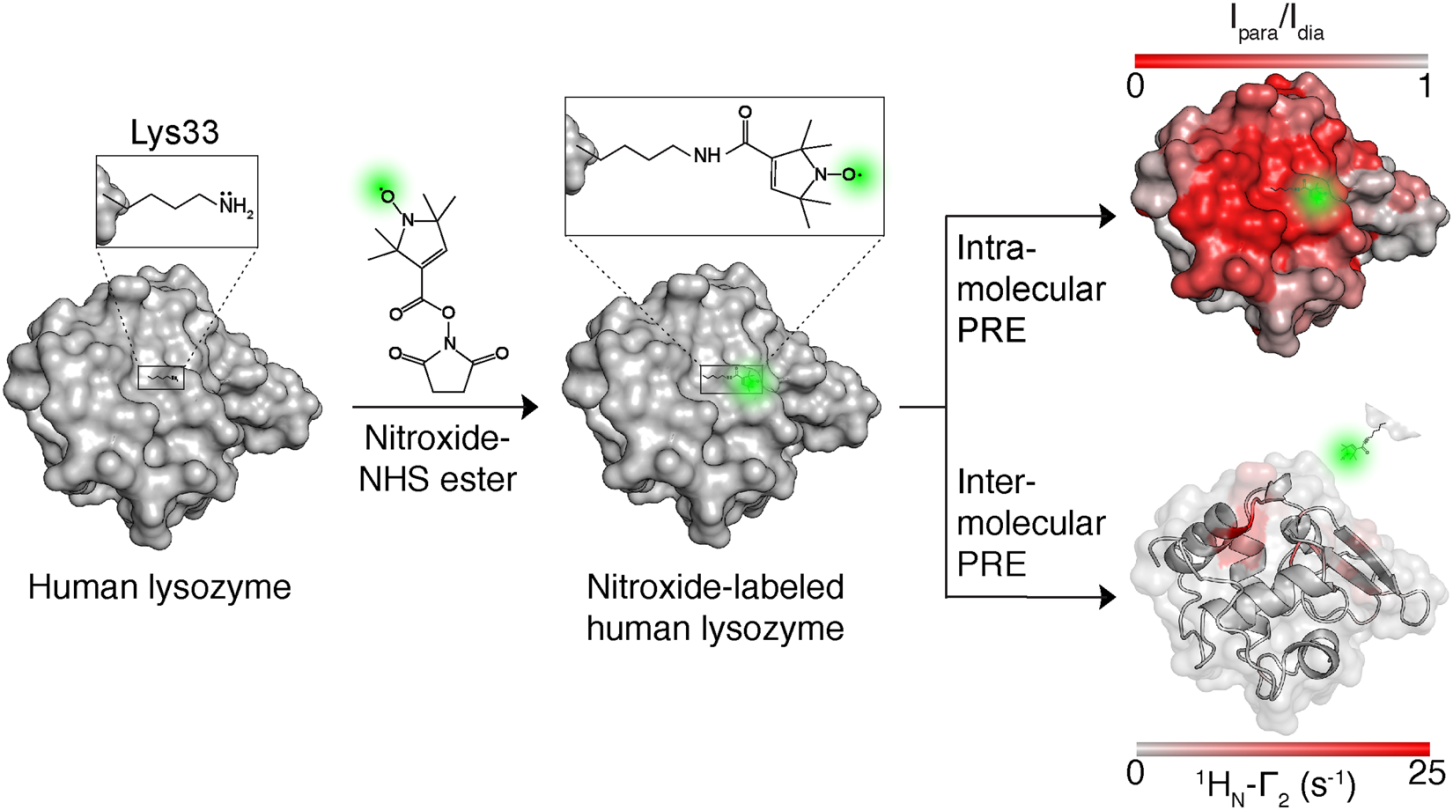
Human lysozyme is site-specifically labeled with a nitroxide group via a lysine residue. Intra- and intermolecular paramagnetic relaxation enhancement (PRE) was measured by NMR to report on native-state structure, but more importantly on transient intermolecular interactions between partially unfolded protein species. Specific lysine labeling provides an alternative strategy for systems that are not amenable to the use of cysteine-targeting methods.

In this study we have aimed to incorporate nitroxide spin labels using our selective lysine labeling strategy^25^ in order to investigate transient intermolecular interactions between monomeric species present during the early events of lysozyme fibril formation. Intramolecular PRE experiments of spin-labeled I59T lysozyme variants, under native conditions, were used to confirm the functionality and applicability of PRE NMR experiments (Fig. 1). These spin-labeled proteins were further studied, under fibril forming conditions, and intermolecular PREs between transiently populated species were observed, demonstrating that the earliest interactions occur between the unfolded β-domain region of the non-native species.

## Results

### WT human lysozyme is spin-labeled at K33

**Figure 2:**
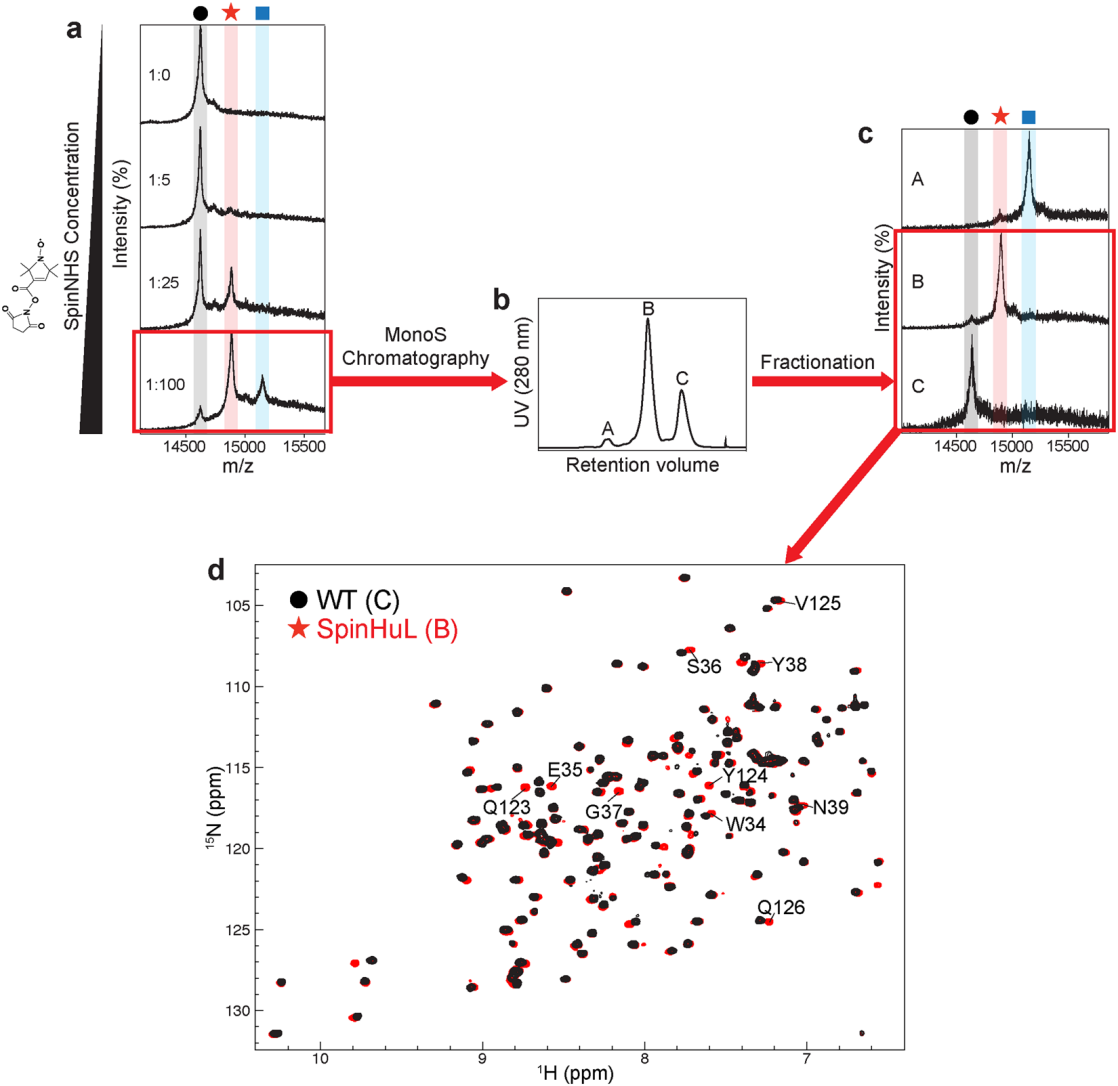
Site-specific nitroxide spin labeling of WT human lysozyme at K33. (**a**) MALDI MS analysis of the effects of SpinNHS at different concentrations on the labeling reaction. The black circle, red star and blue square represent WT and spin-labeled WT human lysozyme with single and double nitroxide labeling (molecular weights of 14,694, 14,860 and 15,026 Da, respectively). Samples were incubated with 7 μM WT human lysozyme and different SpinNHS concentrations (1:0, 1:5, 1:25 and 1:100 lysozyme-to-SpinNHS molar ratio) in MES buffer (0.1 M, pH 5) at 20 °C for 20 hrs. (**b**) Elution profile of the 1:100 labeling reaction from MonoS cation exchange chromatography. (c) MALDI MS analysis of the samples in (**b**). The black circle, red star and blue square represent the same proteins as in (**a**). (**d**) Overlaid HSQC spectra of sample B (red, reduced by sodium ascorbate) and C (black) from (**c**) recorded at pH 5.0, 37 °C and 500 MHz. Amide protons of residues with noticeable chemical shift perturbations are labeled.

Previously, we reported selective biotin labeling of human lysozyme at the most reactive of its five lysine residues (K33) using an active ester (N-hydroxysuccinimide, NHS) at relatively low pH (pH 5). Here we have extended our labeling method by introducing a nitroxide to K33 of WT lysozyme for PRE measurements. At a molar ratio of 1:100 (lysozyme-to-SpinNHS (1-oxyl-2,2,5,5-tetramethylpyrroline-3-carboxylate N-hydroxysuccinimide ester)), the majority of WT lysozyme was labeled with a single nitroxide as shown in Fig. 2a. The single, double and non-labeled proteins could be separated by cation exchange chromatography on a MonoS^®^ column, as the protein becomes less positively charged when the ε-amino group is labeled with the nitroxide moiety (Fig. 2b). Analysis by mass spectrometry of the samples collected from each elution peak confirms the identity of the labeled species (Fig. 2c).

HSQC spectra of WT human lysozyme and the WT containing a single nitroxide (SpinHuL) were recorded and are shown in Fig. 2d. The assignment of the SpinHuL protein was confirmed by a HNCA experiment (see Supplementary Fig. S1). As with our previously reported selective biotinylation of WT lysozyme at K33^25^, the SpinHuL protein shows only marginal chemical shift perturbation of amide peaks (Δδ) in a few residues that are close to K33, either in amino acid sequence or in space, confirming that the overall native fold of the WT protein is retained (Supplementary Fig. S1).

### Intramolecular PREs confirm the location and functionality of the nitroxide spin label on the lysine side chains

To demonstrate that the nitroxide labeling is amenable to measuring PREs we recorded intramolecular PREs on the native state of WT lysozyme at pH 5 and 37 °C (Supplementary Fig. S2). The HSQC spectrum of the paramagnetic sample was recorded, after which the sample was reduced by sodium ascorbate and used directly to record a subsequent HSQC spectrum of the diamagnetic sample (see Supplementary Fig. S2). The reduced sample was preferred over non-labeled protein as the diamagnetic sample, as observing the same protein after reduction avoids introducing any unwanted structural and biophysical differences between the spin-labeled and non-labeled protein samples. The effect of the addition of sodium ascorbate to the total volume of the sample was minimal (<0.5%) and taken into account when calculating the intensity ratios (I_para_/I_dia_). The I_para_/I_dia_ of the SpinHuL was calculated from the HSQC spectra and mapped onto the structure of the protein (Supplementary Fig. S2). A significant number of residues experience PRE effects due to close proximity to the K33 spin label, either in amino acid sequence (residues 30–40) or in the native fold (N-terminus and the region near residue 125) (Supplementary Fig. S2), confirming the location of the spin label at K33 and its utility for PRE experiments.

In order to enhance the chances of detecting intermolecular interactions between intermediate species that are produced only at low concentrations, we used the well-characterized I59T human lysozyme for the PRE experiments as this variant is able to populate the intermediate species more readily than the WT^8,26^. A nitroxide spin label was introduced to K33 of I59T lysozyme and to obtain more information from the PRE experiments spin labels were included at two additional sites. The labeling of other lysine residues was achieved using an I59T variant containing a Lys33–to-Arg33 mutation (I59T-K33R) which removes the most reactive lysine residue. The I59T-K33R variant shows effectively identical biophysical characteristics to the I59T variant (see Supplementary Fig. S3 and Supplementary Table S1), and can be labeled with a single nitroxide spin label at either K13 (K13spin) or K97 (K97spin), as the reactivity of these two lysine residues appear similar. Despite the fact that K13spin and K97spin would be expected to have similar pIs, we observed two discernable peaks when the reaction mixture was separated using a MonoS cation exchange column (see Supplementary Fig. S4), indicating that the location of the spin label influences the affinity of the protein for this ion exchange resin. Although K13spin and K97spin eluted at 24 and 26 min respectively, there was significant overlap in the chromatogram (Supplementary Fig. S4); fractions were, however, carefully collected to ensure sample purity (Supplementary Fig. S4) and the HSQC spectra of the paramagnetic and diamagnetic samples of these proteins were recorded and are shown in Supplementary Fig. S4. The native fold of I59T was retained in both proteins, as in I59T with a nitroxide label at K33, and each protein shows a high level of purity (>90% and >95% for K13spin and K97spin, respectively).

**Figure 3:**
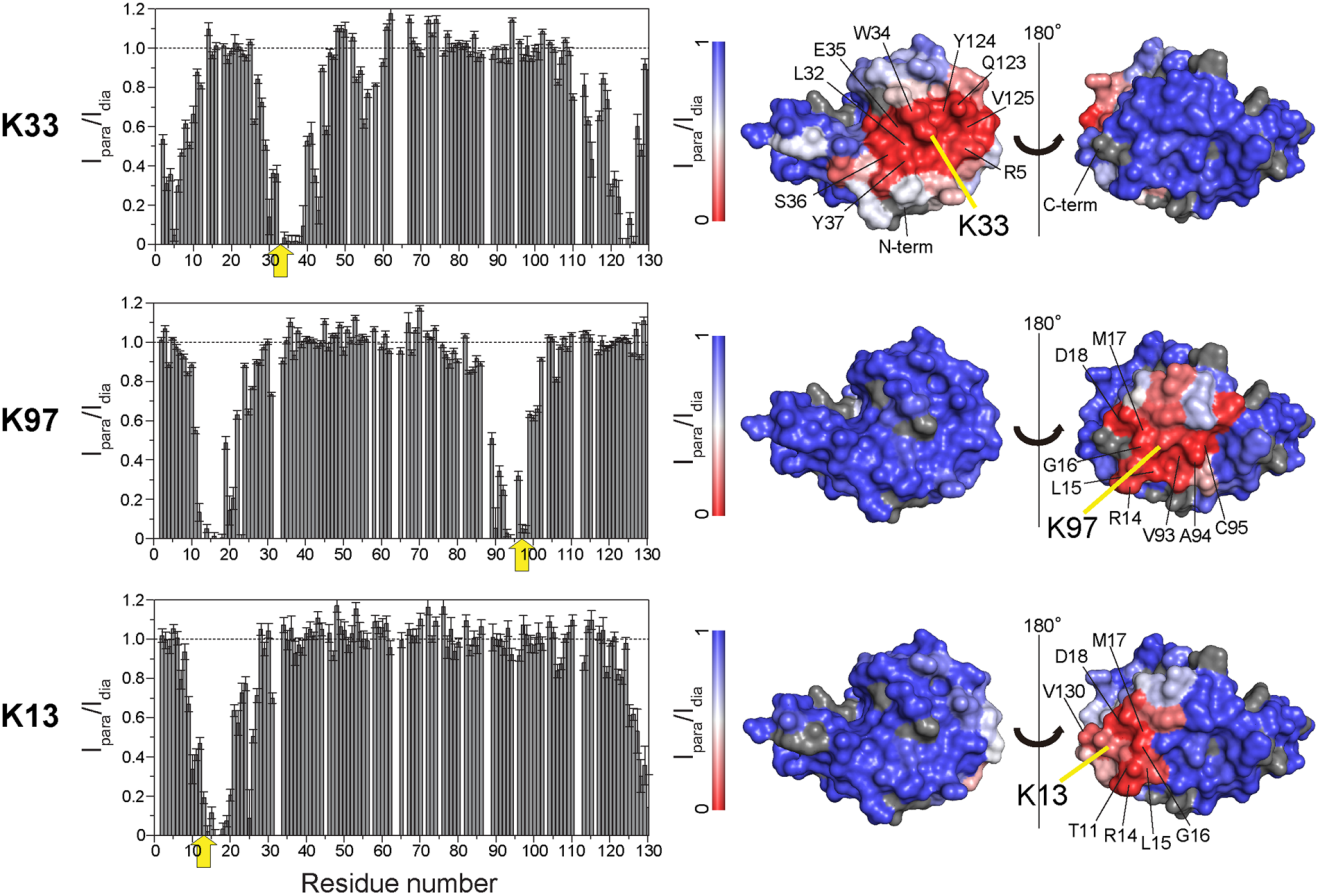
Intramolecular PRE effects induced by nitroxide-spin labels at three individual lysine residues (K33 in I59T, K13 or K97 in the I59T-K33R) at pH 5, 37 °C. Intensity ratios of the correlation peaks in the paramagnetic and diamagnetic samples were measured and used for mapping the structures. The intensity ratios that appear higher than 1.0, due to experimental error, are mapped as 1.0. Low and high PRE effects are shown in blue and red, respectively. Residues where PRE values could not be measured are shown in dark grey and the yellow arrows indicates the location of the nitroxide spin labels. The intramolecular PREs of natively folded I59T with the nitroxide label at K33 show an effectively identical PRE profile to the K33 spin labeled WT protein (see Supplementary Fig. S2). The PRE effects from spin labels located at K97 and K13 relate residues which are close to the spin label either in the amino acid sequence (residues 85–105 for K97; residues 5–30 for K13) or in spatial terms (residues 10–25 for K97; residues 125–130 for K13).

The location and the functionality of the spin label at the three individual lysine residues were confirmed by measuring intramolecular PREs within the native states (pH 5) (Fig. 3), which showed the decrease in the intensity ratios of a number of residues located in distinct positions in the sequence relative to the position of each spin label.

### Intermolecular PREs are observed from non-native forms of the I59T variant present at the thermal denaturation temperatures

**Figure 4:**
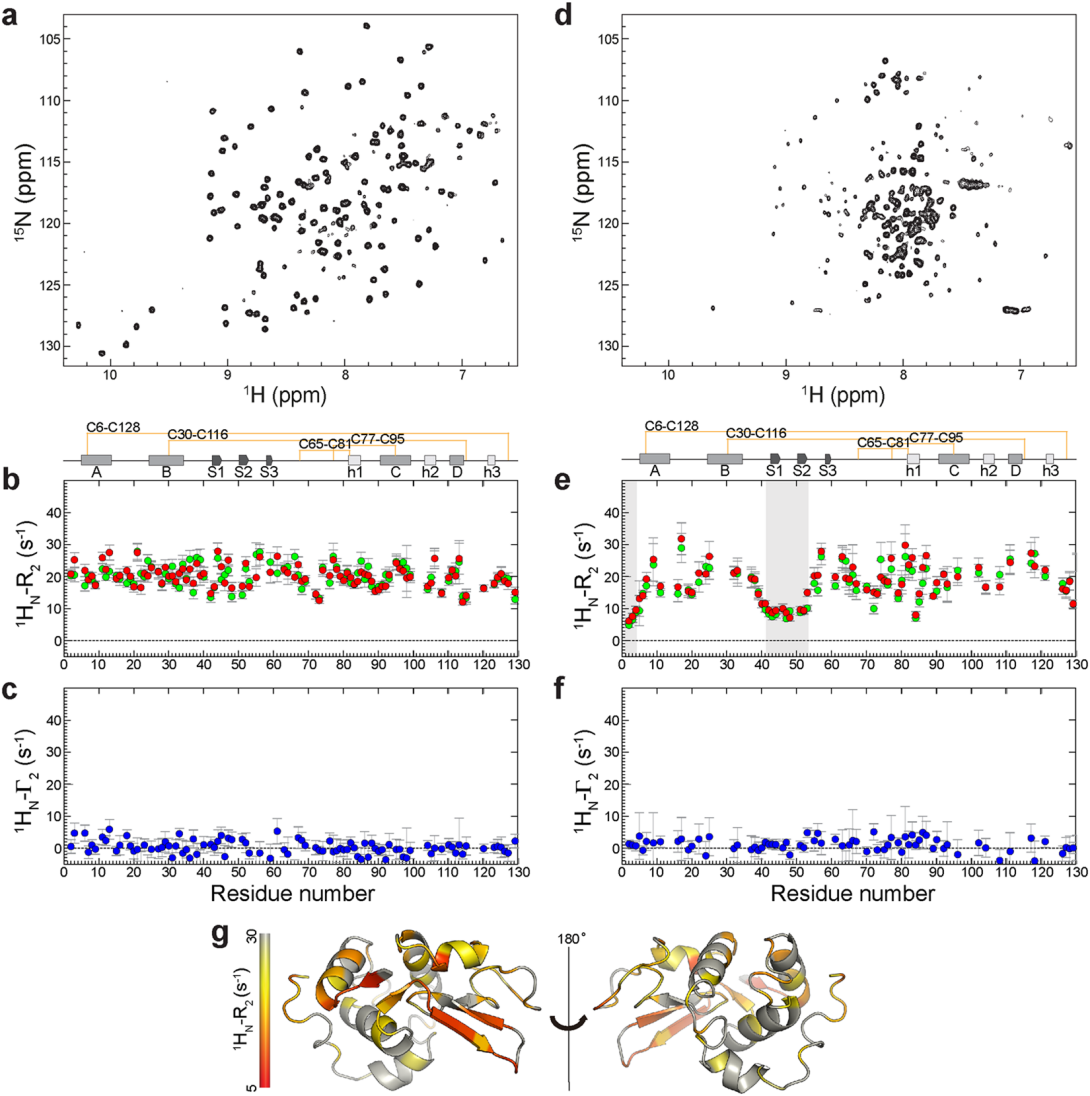
Intermolecular PRE relaxation experiments for WT lysozyme (40 and 50 °C, 500 MHz) at pH 1.2. Spin-labeled human lysozyme and ^15^N-isotopically labeled protein were mixed to give a 1:1 molar ratio and R_2_ relaxation experiments were performed for both paramagnetic and diamagnetic samples. Eight relaxation delay times (8, 13, 18, 23, 28, 38, 48 and 68 ms) were used to collect ^1^H_N_-R_2_ data. (**a**–**c**) HSQC spectrum of WT (**a**), ^1^H_N_-R_2_ (**b**) and ^1^H_N_-Γ_2_ (**c**) of the native state peaks at 40 °C. (**d**–**f**) HSQC spectrum of WT (**d**), ^1^H_N_-R_2_ (**e**) and ^1^H_N_-Γ_2_ (**f**) of the unfolded state peaks at 50 °C. (**g**) Structure of WT colored by ^1^H_N_-R_2_ values measured from the unfolded state peaks in (**e**). Red and green data points in (**b**,**e**) indicate ^1^H_N_-R_2_ of the paramagnetic and diamagnetic samples. Red coloring on the lysozyme structure in (**g**) indicates the regions of the protein that are flexible and predominantly unfolded as indicated by low ^1^H_N_-R_2_ values.

To detect potential intermolecular interactions between the transient intermediate states in the early stages of lysozyme fibril formation, intermolecular PREs were first measured using SpinHuL and ^15^N-labeled WT lysozyme. The ^1^H_N_-Γ_2_ values of the WT protein were calculated using the difference between ^1^H_N_-R_2_ values of the paramagnetic and diamagnetic samples (Fig. 4) in order to circumvent potential limitations of the intensity ratio method, which can also be affected by other factors such as ^1^H_N_-Γ_1_ and coherence transfer delay^12,27^.

Overall, no significant intermolecular PREs were detected for the native or the unfolded states at 40 and 50 °C (Fig. 4c and f). This indicates that the non-native protein species which are postulated to experience intermolecular interactions, thus resulting in PRE effects, are either too scarce to give rise to detectable Γ_2_, or the value of Γ_2_ in the complex is itself very small. One interesting feature is present in the R_2_ values of the unfolded states at 50 °C (Fig. 4e), in that some residues, such as 1–3 and 41–52, show noticeably reduced ^1^H_N_-R_2_ values (<10 s^−1^), as expected for an unstructured or denatured protein. In contrast, other regions of the unfolded state show faster R_2_ rates in a similar trend to that of the R_2_ rates of HEWL^28,29^, where the unstructured parts of the protein show slow R_2_ rates in contrast to the other regions that have faster R_2_ rates as a result of residual hydrophobic structures and long range intramolecular interactions mediated by the presence of disulfide bonds under oxidized conditions. Likewise, our data suggest that the residues with slower R_2_ rates are likely to be more flexible in the unfolded state due to the partial unfolding of this region, whereas other regions show faster R_2_ rates potentially because of the presence of the disulfide bonds that are maintained in the unfolded state. Indeed the residues with slower R_2_ rates correspond to those in the S1 and S2 strands of the β-sheet (41–52), which unfold first as the temperature increases at the same pH in the I56T variant^8^. Also, the residues that display fast R_2_ rates are near the eight cysteine residues that form disulfide bonds within the native state. Therefore, despite the absence of detectable PREs under these conditions in the WT human lysozyme, relaxation experiments allow structural insights, indicating that the unfolded state is not fully unstructured, but maintains some residual structures likely due to the presence of the four disulfide bridges.

**Figure 5:**
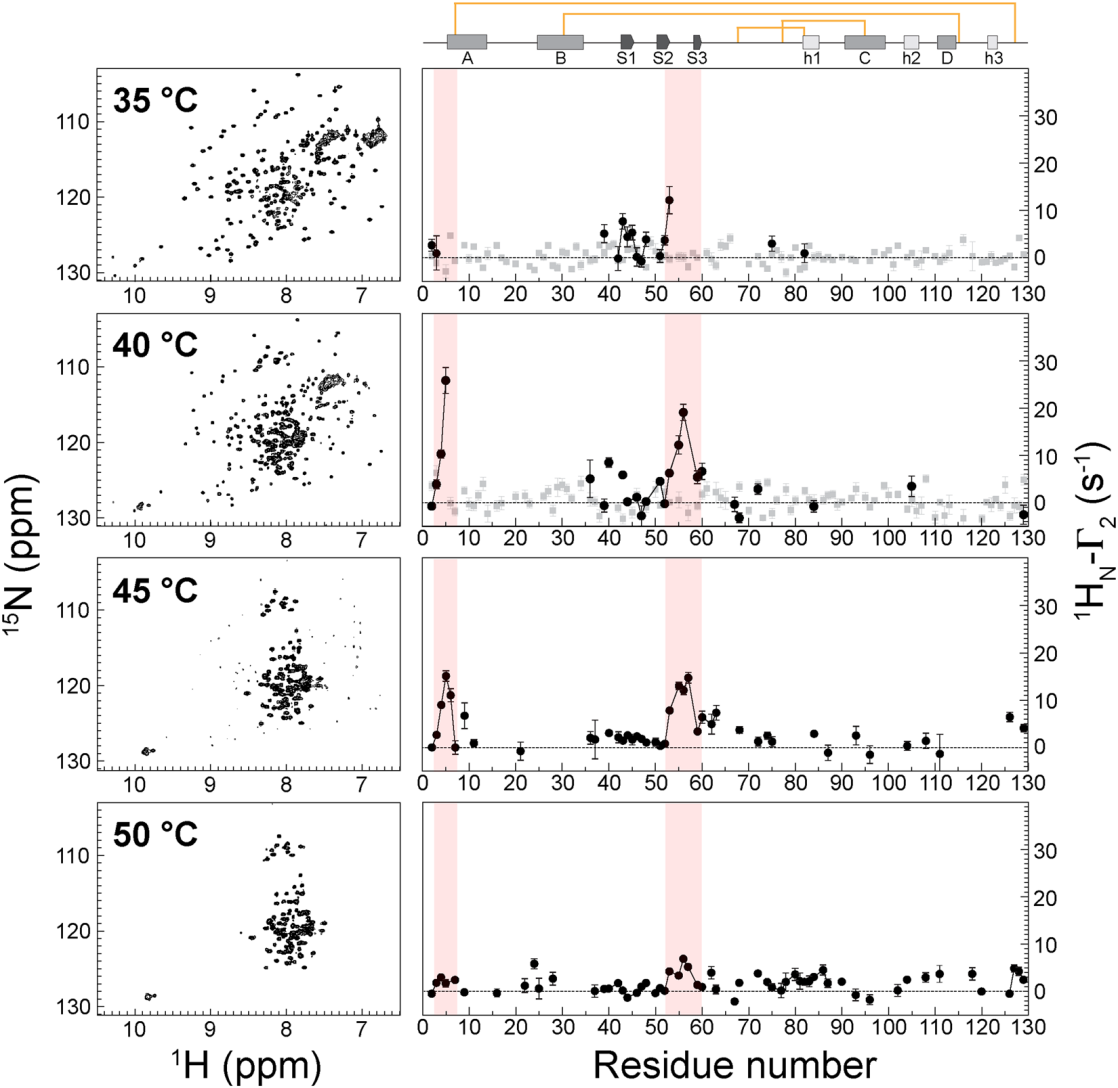
Temperature evolution of the intermolecular PREs for I59T lysozyme (pH 1.2, at 35, 40, 45 and 50 °C) with a K33 nitroxide spin label. HSQC spectra of the diamagnetic sample at each temperature are shown on the left. The spectra at 25 and 45 °C with assignments are shown in Supplementary Figs S5 and S6, respectively. ^1^H_N_-Γ_2_ values (defined as the difference between ^1^H_N_-R_2, para_ and ^1^H_N_-R_2, dia_) were calculated from ^1^H_N_-R_2_ values measured using two relaxation delay times (7, 37 ms) at 700 MHz. Red shading indicates regions of the N-terminus and the small loop at the domain interface where noticeable PREs are observed. Grey squares and black circles represent ^1^H_N_-Γ_2_ values measured from native and non-native peaks, respectively.

In order to increase the population of the intermediate state species, thereby increasing the chances of detecting any intermolecular interactions, intermolecular PREs were measured for the I59T variant. At pH 1.2, I59T shows a larger intermediate state population than the WT protein due to lower native stability and a loss of global cooperativity^8^. To minimize the chances of inducing unwanted denaturation of the protein via acid-hydrolysis, we chose to use two relaxation delays instead of eight, thereby reducing the duration of the experiment, as it is possible to measure relaxation rates with high accuracy by using two relaxation delays^20^. Intermolecular PREs were measured on the ^15^N-labeled I59T human lysozyme in the presence of the same protein containing a K33 spin label using pH 1.2 at different temperatures (35, 40, 45 and 50 °C) (Fig. 5).

The native peaks give rise to no significant ^1^H_N_-Γ_2_ values at low temperatures; however, a large number of non-native state residues were found to have measurable ^1^H_N_-Γ_2_ values at higher temperatures with their peak intensities increasing when the protein progressively unfolds. Moreover, two regions of the protein (the N-terminus and the domain interface) show noticeable PREs that were not observed within the more stable WT protein (Fig. 4). The magnitudes of the ^1^H_N_-Γ_2_ values reach a maximum at 40 °C, then decrease with further temperature increases as the population of the conformational species giving rise to these PRE decreases. Intramolecular PRE measurements on K33 spin labeled WT and I59T (see Supplementary Figs S7 and S8) under similar conditions suggest that the non-native protein species possess a significant degree of residual structure. Analysis of the thermal unfolding of I59T monitored by far- and near-UV circular dichroism (CD) spectroscopy predicts that the maximum population of the intermediate state should exist at ca. 40 °C, based on a three-state unfolding model^8^. Overall the data suggest that the ultra-weak protein interactions^14^, observed in the intermolecular PREs, have resulted from lysozyme monomers that are distinct from the native-state, but still retain residual compact structures similar to the partially unfolded protein species that are crucial in lysozyme fibril formation.

### Intermolecular PREs from three different spin labels (K13, K33 and K97) report on interactions involving the unfolded β-domain

**Figure 6:**
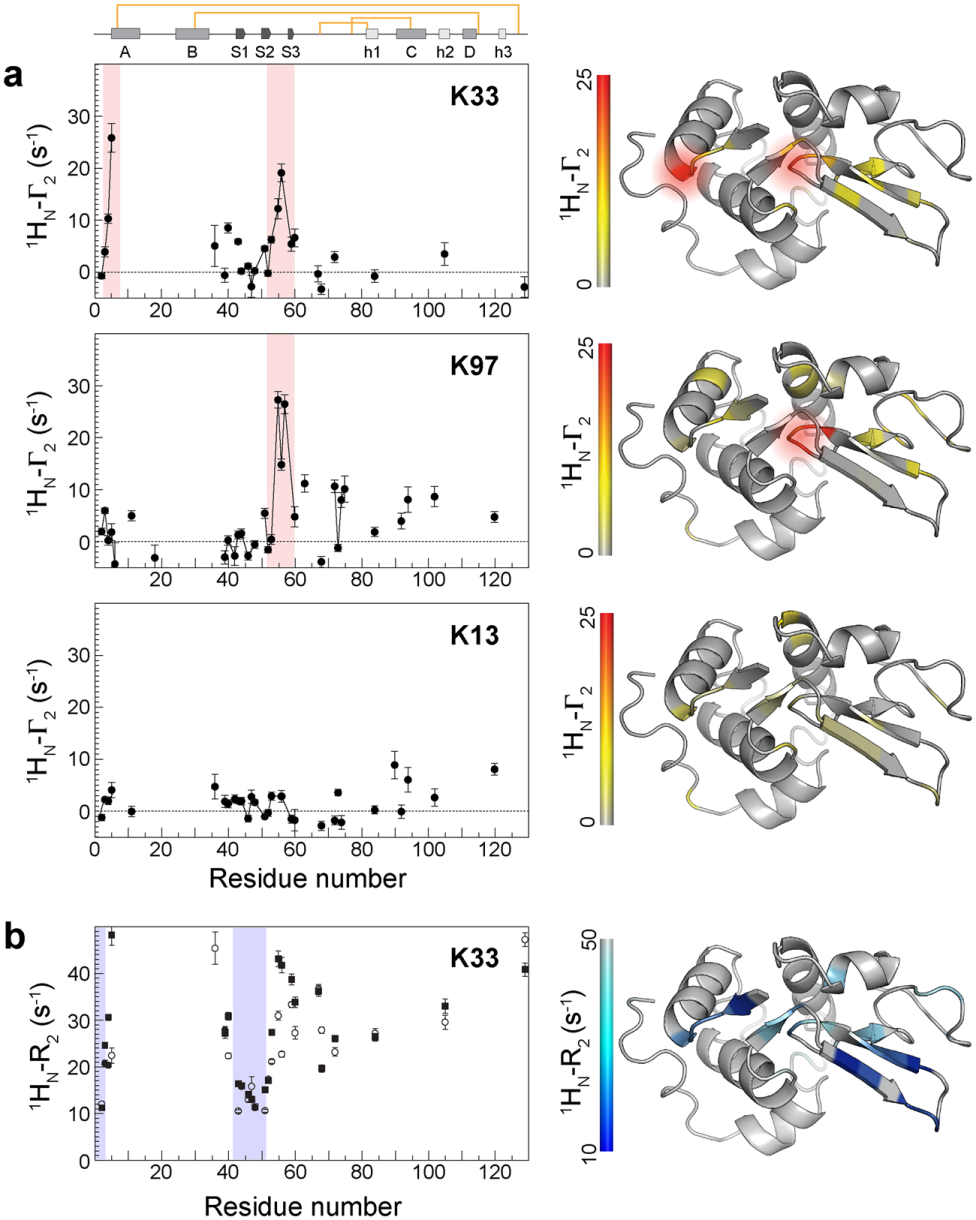
(**a**) Intermolecular PREs for I59T lysozyme (pH 1.2, 40 °C) with spin labels at three different locations: K33, K97 and K13. ^1^H_N_-Γ_2_ values were calculated from the ^1^H_N_-R_2_ values measured using two relaxation delay times (7, 37 ms) at 700 MHz and used for mapping the structures. Red shading indicates regions of the N-terminus and the small loop at the domain interface where noticeable PREs are observed as in Fig. 3. (**b**) ^1^H_N_-R_2_ of I59T with spin label at K33 at 40 °C and the structure colored by ^1^H_N_-R_2_ of the diamagnetic sample. Filled squares and open circles indicate ^1^H_N_-R_2_ of the paramagnetic and diamagnetic samples, respectively. Data points from diamagnetic samples are used for mapping the structure. Blue shading indicates regions of the N-terminus and the β-hairpin regions where low ^1^H_N_-R_2_ values are observed (<15 s^−1^).

In order to obtain additional structural information on the intermolecular interactions, PREs induced by K13spin and K97spin were measured at the optimized temperature of 40 °C (Fig. 6a). The results clearly show that the spin labels at different locations generate distinct PRE profiles; the K97spin shows PRE values only in the loop region at the domain interface (residues 55–57), whereas the K13spin shows no significant PREs. The effects are not due to non-specific solvent PREs, as the same molar ratio of free nitroxide molecules added to I59T lysozyme does not show any PRE effects (data not shown). It is interesting to note that analysis of ^1^H_N_-R_2_ values for the K33spin protein shows that the regions adjacent to those displaying intermolecular PREs have reduced relaxation rates, suggesting a potential interplay of these two neighboring regions (Fig. 6b).

## Discussion

Central to specific spin labeling of protein molecules for PRE measurement by NMR has been the use of site-directed mutagenesis to substitute a desired amino acid with a single cysteine residue, which can subsequently be reacted with thiol-reactive reagents, such as MTSL^18^. This strategy is advantageous as solvent-exposed cysteines can be readily labeled without inducing side reactions. It has some limitations, however, in cases where the naturally existing cysteines have critical structural (i.e. disulfide bridges) or functional (i.e. acting as a ligand or a catalytic center in various reactions) roles and cannot be mutated into serine or alanine^30^. For such systems our approach of selectively introducing nitroxide labels at lysine residues via a nucleophilic acyl substitution reaction is facile and offers an alternative labeling strategy avoiding interference with naturally existing cysteine residues. As this selective lysine-targeting method hinges on different reactivities of lysine based on their pKa values, it also has limitations as it is not uncommon to find a protein that contains multiple lysine residues with similar mobility and accessibility to the solvent. In such situations, however, a number of other labeling strategies can be applied, such as total-synthesis^17^, tag-promoted complexation^31^ or direct biosynthesis^32^. These methodologies require a greater degree of protein engineering; however, enable highly specific introduction of the desired probes.

When the native state (N) of lysozyme unfolds, it populates an ensemble of partially denatured species (D) that are transient and heterogeneous^8^. Under such conditions there can be three types of intermolecular interactions between these species: N-N, N-D and D-D. The first and second type of interactions do not contribute to the observed PRE effects as no intermolecular PREs are detected for native state residues; therefore, the observed intermolecular PREs arise solely from the interactions between the species that form the denatured ensemble. Given that the magnitude of the intermolecular PREs from protein conformers is small and decreases with increasing temperature, it is probable that the PREs are detected from a specific type of partially unfolded species that is only marginally and transiently populated near the melting temperature.

**Figure 7:**
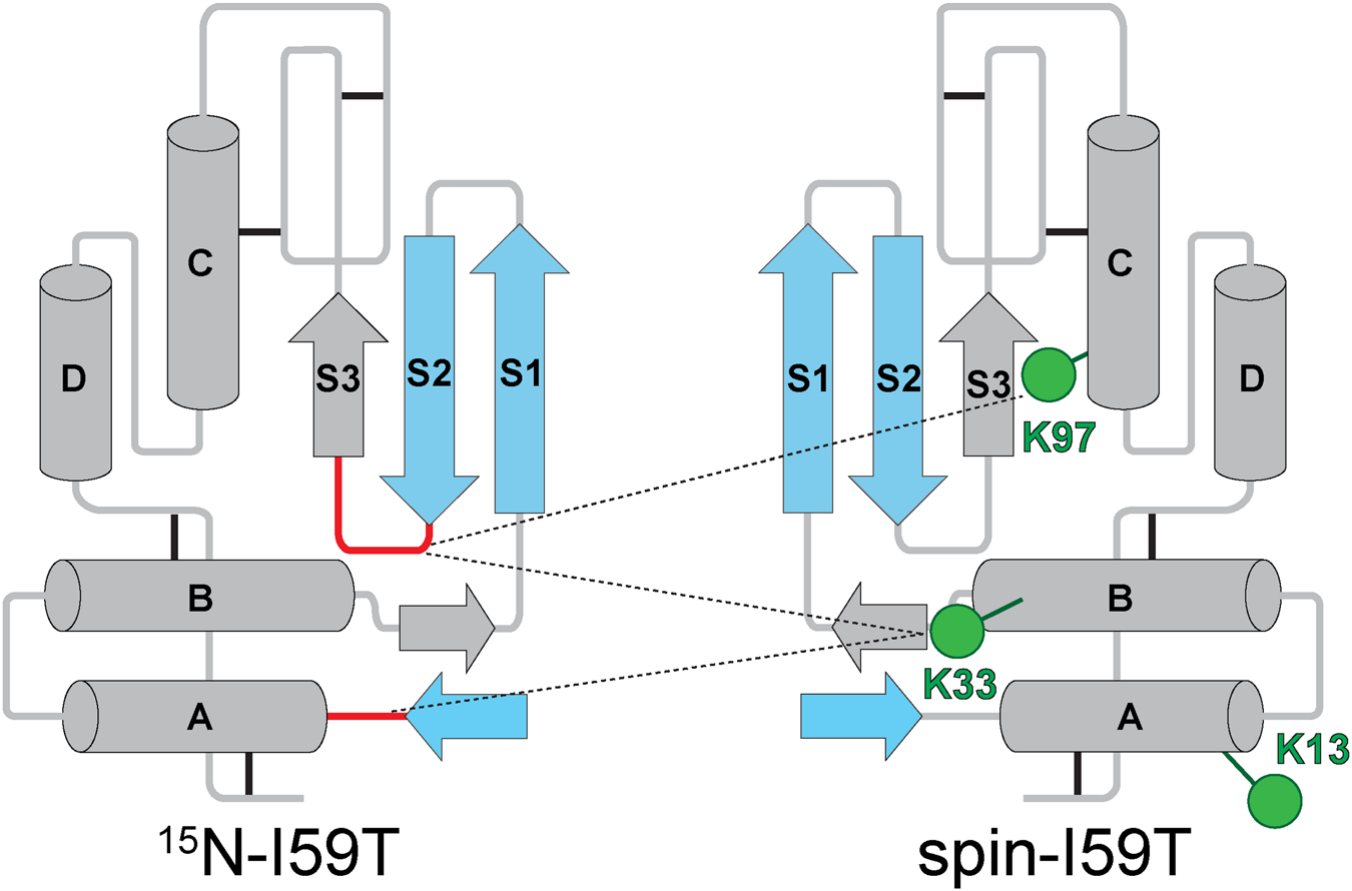
Schematic diagram of the proposed unfolded β-domain region involved in early intermolecular interactions of I59T lysozyme. The structural elements shown in blue represent the regions that are predominantly unstructured and flexible (^1^H_N_-R_2_ < 10 s^−1^) (Fig. 3(b)), whereas the regions experiencing high PREs (^1^H_N_-Γ_2_ > 10 s^−1^) are shown in red. Green circles mark the nitroxide spin labels that are located at the three lysine residues (K13, K33 and K97, Fig. 3(a)). The black lines indicate the four disulfide bonds. Dashed lines indicate the potential intermolecular contacts giving rise to significant ^1^H_N_-Γ_2_ values.

The gradual appearance of the unfolded state peaks in the HSQC spectra of human lysozyme with increasing temperature reveals that at 40 °C, residues at the N-terminus (residues 1–8), in the β-hairpin of the native state (residues 41–52) and the small loop at the domain interface (residues 55–59) are predominantly unstructured, whilst the rest of the protein retains highly native-like structure. In addition, the residues at the N-terminus and β-hairpin show reduced ^1^H_N_-R_2_ values (<10 s^−1^) in both the WT and I59T proteins, representative of flexibility that is close to that of a completely random coil structure (Fig. 3). Under these conditions the spin labels that are close to the β-sheet region (K33 and K97) display distinct PRE signals, whilst the spin label that is spatially distant from this region (K13) shows little or no effects. Using the results obtained from our experiments, we illustrate the interactions in a schematic diagram shown in Fig. 7. Based on this information we conclude that in the partially unfolded lysozyme state, initial intermolecular contacts are made through interactions involving the unfolded region of the structure that, in its native state, forms the β-domain, and these interactions are consistent with the proposed aggregation model of human lysozyme, which identifies the population of the partially unfolded intermediate species as crucial to amyloid fibril formation^6,33^.

## Conclusion

We have demonstrated that transient protein interactions can be determined by PRE NMR measurements using the selective lysine labeling strategy discussed in this study. This strategy could be widely applied to other systems, including those where more conventional site-directed engineering to introduce single cysteine residues for labeling is not practical, as modification of lysine residues does not usually alter the chemical nature of a protein due to the high solvent-accessibility and mobility of these residues^34,35^. By utilizing a strategy to attach spin-labels specifically to individual lysine residues in human lysozyme, we have been able to capture the early intermolecular interactions between non-native I59T lysozyme molecules that are likely to trigger the subsequent aggregation process which leads to amyloid deposition in patients possessing pathological mutations in their lysozyme gene.

## Methods

All chemicals and reagents were purchased from Sigma Aldrich Ltd. (Gillingham, UK) unless otherwise stated.

### Preparation of I59T-K33R plasmid

Site-directed mutagenesis to introduce K33R was performed on pPIC9 containing the I59T human lysozyme gene using the Quick Change Site-Directed Mutagenesis protocol (Agilent Technologies, Oxford, UK). The K33R mutation was confirmed by DNA sequencing, performed at the Sequencing Facility in the Department of Biochemistry, University of Cambridge.

### Site-specific modification of lysine side-chains of human lysozyme with nitroxide label

The WT, I59T and I59T-K33R human lysozymes were expressed in *Pichia pastoris* and purified as previously described^36,37^. In order to produce isotopically labeled samples for NMR experiments, ^15^N ammonium sulfate was used to label the protein produced in *P*. *pastoris* as previously described^37^. The level of isotope labeling was assessed by MALDI mass spectrometry and found to be essentially complete (>99%).

K33 of WT and I59T human lysozymes was labeled with nitroxide by incubating the protein with 1-oxyl-2,2,5,5-tetramethylpyrroline-3-carboxylate N-hydroxysuccinimide ester (SpinNHS) at a lysozyme-to-SpinNHS molar ratio of 1:100 (WT) and 1:300 (I59T) using the same reaction conditions as those reported for biotinylation of WT lysozyme^25^. Mass spectrometry of the samples at the endpoint of the reactions confirmed that the predominant species (80–90%) was singly-labeled lysozyme. Dialyzed samples were flash frozen in liquid nitrogen and lyophilized. Nitroxide spin-labeled WT and I59T samples were dissolved in Tris buffer (50 mM, pH 8) and purified using a MonoS^®^ ion exchange column (GE Healthcare, Amersham, UK) on an ÄKTA Pure purification system (GE Healthcare). The protein was eluted using a sodium chloride gradient (0–1 M) and multiple fractions were collected. The fractions containing protein were dialyzed against deionized water (dH_2_O) (2 × 4 h and 24 h) or centrifuged (4 × 10 min, 15,000 g, dH_2_O was replenished to the filter unit after each repetition) in an Amicon^®^ Ultra-0.5 centrifugal filter unit (3,000 nominal molecular weight limit, 500 μL, Millipore, Watford, UK) to remove the remaining sodium chloride. Samples in dH_2_O were flash frozen in liquid nitrogen, lyophilized and used for further analysis by dissolving in the respective buffers. Mass spectrometry of the single spin-labeled sample after the purification indicated that the sample was pure and homogeneous (>99%, Figure S1(b)). K97 and K13 of I59T-K33R were spin-labeled in the same way as described above using a higher lysozyme-to-SpinNHS molar ratio (1:500). The sample after incubation was purified in the same way on the MonoS^®^ ion exchange column. Interestingly, this reaction yielded two populations that were resolved as separate peaks on the chromatogram (retention times at 24 and 26 min, respectively) for spin labeled K13 and K97; with the latter being the predominant species. Collection of the individual peaks resulted in the expected mass for each species, and high purity of each species (>95 and >90% for K97 and K13 spin-labeled lysozyme, respectively) was confirmed by HSQC NMR spectra.

### Circular dichroism spectroscopy

CD spectroscopy experiments were performed using a Jasco J-810 spectropolarimeter (JASCO Ltd, Great Dunmow, UK) equipped with a Peltier temperature controller. Protein samples (20 μM) were dissolved in sodium citrate buffer (10 mM, pH 5) and analyzed in a 0.1 cm cuvette. Thermal denaturation was monitored at 222 or 270 nm with temperatures increasing from 5 to 95 °C (1 °C min^−1^). Ellipticity values were normalized to the fraction of unfolded protein (F_u_) using equation 1:

1 $${{\rm{F}}}_{{\rm{u}}}=\frac{({\rm{\theta }}-{{\rm{\theta }}}_{{\rm{N}}})}{({{\rm{\theta }}}_{{\rm{U}}}-{{\rm{\theta }}}_{{\rm{N}}})}$$

where θ is the observed ellipticity, and θ_N_ and θ_U_ are the ellipticities of the native and the unfolded states, respectively. θ_N_ and θ_U_ were extrapolated from pre- and post-transition baselines. Experimental data were fitted to a two-state unfolding model assuming linear baselines for both native and unfolded states using equation 2:

2 $${\rm{S}}=\frac{{({\rm{S}}}_{{\rm{N}}}+{{\rm{m}}}_{{\rm{N}}}{\rm{T}})+({{\rm{S}}}_{{\rm{U}}}+{{\rm{m}}}_{{\rm{U}}}{\rm{T}}){{\rm{e}}}^{(-\frac{{\rm{\Delta }}{\rm{G}}}{{\rm{RT}}})}}{1+{{\rm{e}}}^{(-\frac{{\rm{\Delta }}{\rm{G}}}{{\rm{RT}}})}}$$

where S_N_, S_U_ are the signals for native and unfolded states at T = 0, whilst m_N_ and m_U_ are the slopes of the baseline for native and unfolded states, respectively. ΔG represents the change of the free energy for the transition. The fitting was performed by OriginPro 8.0 (OriginLab Corporation, Northampton, MA, USA) and the mid-point T_m_ values are defined as the temperatures where F_u_ is 0.5.

### Thermal denaturation monitored by 8-anilino-1-napthalenesulfonic acid (ANS) fluorescence emission

Thermal denaturation monitored by ANS fluorescence emission was recorded on a Cary Eclipse spectrofluorimeter (Agilent Ltd., Oxford, UK) using excitation and emission wavelengths of 350 and 475 nm, respectively (slit-widths 5 nm) with increasing temperatures from 20 to 95 °C (0.5 °C min^−1^). Samples contained 2 μM protein (0.1 M sodium citrate (pH 5.0), 360 μM ANS). Transition curves of ANS alone (360 µM) were collected and subtracted from all samples. The fluorescence data were normalized with respect to the ANS emission spectrum in the presence of the I56T variant. Experimental data were fitted to a Gaussian expression using OriginPro 8.0

### Hydrogen/deuterium exchange (HDX) monitored by mass spectrometry

EX1 HD exchange of I59T and I59T-K33R variants were monitored as previously described^38^.

### Lysozyme aggregation monitored by thioflavin-T binding

Aggregation studies were performed in triplicate with lysozyme variants (6.8 μM, 0.1 M sodium citrate buffer, pH 5.0, 62.5 μM thioflavin-T (ThT)) incubated with stirring at 60 °C in a Cary Eclipse spectrofluorimeter. ThT fluorescence was measured with excitation and emission wavelengths of 440 nm and 480 nm (slit-widths 5 nm) respectively.

### Transmission Electron Microscopy (TEM)

Samples for TEM were prepared on carbon support film, 400 mesh, 3 mm copper grids (EM Resolutions Ltd., Saffron Walden, UK) and stained with 2% uranyl acetate (w/v). The samples were imaged on a FEI Tecnai G2 transmission electron microscope in the Cambridge Advanced Imaging Centre (CAIC, University of Cambridge, Cambridge, UK). Images were analyzed using the SIS Megaview II Image Capture system (Olympus, Tokyo, Japan).

### NMR spectroscopy

The backbone resonances of the isotopically double-labeled (^13^C, ^15^N) nitroxide spin-labeled lysozyme (SpinHuL) were assigned at 37 °C using HNCA experiments^39^. Double-labeled SpinHuL (100 μM) was dissolved in sodium acetate buffer (20 mM, pH 5.0) containing a 90% H_2_O/10% D_2_O mixture. The experiment had 1792 × 48 × 128 complex points (^1^H × ^15^N × ^13^C) with spectral widths of 14 ppm (^1^H), 31 ppm (^15^N) and 40 ppm (^13^C). For reference, HSQC experiments were run with NS = 8 scans in this series of experiments. Spectra were recorded on a Bruker Avance 700 MHz NMR spectrometer (Bruker, Coventry, UK) and processed with NMRPipe^40^ and Sparky^41^. The peak intensities were quantified by FuDA^42^.

Spin-labeled WT human lysozyme (200 μM) was dissolved in sodium acetate buffer (20 mM, pH 5.0) with 90% H_2_O/10% D_2_O and HSQC spectra were recorded at 37 °C on a Bruker Avance 500 MHz NMR spectrometer. Paramagnetic samples were reduced by adding sodium ascorbate (1 mM) and used as the diamagnetic counterparts. Reduction of the paramagnetic samples was monitored by multiple 1D proton and 2D HSQC spectra over time. After full reduction (when there were no further increases in the peak intensities of both 1D and 2D spectra) multiple HSQC spectra with the same parameters for paramagnetic samples were recorded on the diamagnetic samples. The peak heights of the amide protons were measured and the intensity ratios (para/dia) were calculated, these are colored on the crystal structure of the protein (Fig. 1).

^14^N-SpinHuL (250 μM) and ^15^N-WTHuL (200 μM) were mixed in phosphate buffer (50 mM, pH 1.2) with 90% H_2_O/10% D_2_O. ^1^H_N_-R_2_ was measured by relaxation experiments at 40 °C and 50 °C based on published pulse sequences^20,21^ using eight (8, 13, 18, 23, 28, 38, 48 and 68 ms) relaxation delays on a Bruker Avance 500 MHz NMR spectrometer. ^1^H_N_-R_2_ was calculated by fitting a single exponential decay curve to the data. ^1^H_N_-Γ_2_ was calculated by the difference between ^1^H_N_-R_2, para_ and ^1^H_N_-R_2, dia_. The experiment was performed using two relaxation delays (7, 27/37ms) under the same conditions and no noticeable differences were observed in either ^1^H_N_-R_2_ or ^1^H_N_-Γ_2_ values as described and investigated previously^20^. For I59T measurements two relaxation delays were included to reduce the duration of the experiment and the occurrence of unwanted denaturation of the protein via acid-hydrolysis. The ^1^H_N_-Γ_2_ values were measured by performing relaxation-based measurements at 30, 35, 40 and 45 °C on a Bruker Avance 700 MHz NMR spectrometer and using the intensities of the paramagnetic and diamagnetic samples (I_para_ and I_dia_, respectively) with equation 3:

3 $${}^{1}{\rm{H}}_{{\rm{N}}}-{{\rm{\Gamma }}}_{2}=\frac{\mathrm{ln}(\frac{{{\rm{I}}}_{{\rm{dia}},{\rm{Tb}}}\cdot {{\rm{I}}}_{{\rm{para}},{\rm{Ta}}}}{{{\rm{I}}}_{{\rm{dia}},{\rm{Ta}}}\cdot {{\rm{I}}}_{{\rm{para}},{\rm{Tb}}}})}{{{\rm{T}}}_{{\rm{b}}}{-{\rm{T}}}_{{\rm{a}}}}$$

where T_a_ and T_b_ are the two relaxation times^20^. The errors in ^1^H_N_-Γ_2_ (σ) are calculated by the equation 4:

4 $$\sigma ({}^{1}{{\rm{H}}}_{{\rm{N}}}-{{\rm{\Gamma }}}_{2})=\frac{{\{{(\frac{{\sigma }_{{\rm{d}}{\rm{i}}{\rm{a}}}}{{{\rm{I}}}_{{\rm{d}}{\rm{i}}{\rm{a}},{{\rm{T}}}_{{\rm{a}}}}})}^{2}+{(\frac{{\sigma }_{{\rm{d}}{\rm{i}}{\rm{a}}}}{{{\rm{I}}}_{{\rm{d}}{\rm{i}}{\rm{a}},{{\rm{T}}}_{{\rm{b}}}}})}^{2}+{(\frac{{\sigma }_{{\rm{p}}{\rm{a}}{\rm{r}}{\rm{a}}}}{{{\rm{I}}}_{{\rm{p}}{\rm{a}}{\rm{r}}{\rm{a}},{{\rm{T}}}_{{\rm{a}}}}})}^{2}+{(\frac{{\sigma }_{{\rm{p}}{\rm{a}}{\rm{r}}{\rm{a}}}}{{{\rm{I}}}_{{\rm{p}}{\rm{a}}{\rm{r}}{\rm{a}},{{\rm{T}}}_{{\rm{b}}}}})}^{2}\}}^{0.5}}{\text{T}{}_{{\rm{b}}}-\text{T}{}_{{\rm{a}}}}$$

where σ_dia_ and σ_para_ are the standard deviations of the background noise in the spectra for diamagnetic and paramagnetic samples^20^.

Solvent PRE effects that are caused by diffusion and random elastic collision^14,15^ were tested by measuring ^1^H_N_-Γ_2_ values on I59T variant in the presence of equimolar SpinNHS at 40 and 45 °C. No noticeable difference between the ^1^H_N_-R_2_ values of the paramagnetic and diamagnetic sample (after reduction) was observed and all measured ^1^H_N_-Γ_2_ values were effectively zero. 1D (^1^H) and 2D (^1^H-^15^N HSQC or HMQC) experiments were performed before and after all intermolecular PRE measurements and confirmed that there was no degradation or aggregation of the protein. The concentration of the protein samples measured by UV absorbance at 280 nm was also identical before and after the measurements, indicating that no detectable increase in light scattering occurred due to oligomerization or aggregation.

## Electronic supplementary material

Supplementary Information

## References

[1] Dobson CM (2003). Protein folding and misfolding. Nature.

[2] Chiti F, Dobson CM (2008). Amyloid formation by globular proteins under native conditions. Nat. Chem. Biol..

[3] Dumoulin M, Kumita JR, Dobson CM (2006). Normal and aberrant biological self-assembly: Insights from studies of human lysozyme and its amyloidogenic variants. Acc. Chem. Res..

[4] Pepys M (1993). Human lysozyme gene mutations cause hereditary systemic amyloidosis. Nature.

[5] Booth DR (1997). Instability, unfolding and aggregation of human lysozyme variants underlying amyloid fibrillogenesis. Nature.

[6] Canet D (2002). Local cooperativity in the unfolding of an amyloidogenic variant of human lysozyme. Nat. Struct. Biol..

[7] Dumoulin M (2005). Reduced global cooperativity is a common feature underlying the amyloidogenicity of pathogenic lysozyme mutations. J. Mol. Biol..

[8] Dhulesia A (2010). Local cooperativity in an amyloidogenic state of human lysozyme observed at atomic resolution. J. Am. Chem. Soc..

[9] Buell AK (2011). Population of nonnative states of lysozyme variants drives amyloid fibril formation. J. Am. Chem. Soc..

[10] Clore GM (2011). Exploring sparsely populated states of macromolecules by diamagnetic and paramagnetic NMR relaxation. Protein Sci..

[11] Sekhar A, Kay LE (2013). NMR paves the way for atomic level descriptions of sparsely populated, transiently formed biomolecular conformers. Proc. Natl. Acad. Sci. USA.

[12] Clore GM, Iwahara J (2009). Theory, practice, and applications of paramagnetic relaxation enhancement for the characterization of transient low-population states of biological macromolecules and their complexes. Chem. Rev..

[13] Korzhnev DM, Kay LE (2008). Probing invisible, low-populated states of protein molecules by relaxation dispersion NMR spectroscopy: an application to protein folding. Acc. Chem. Res..

[14] Tang C, Ghirlando R, Clore GM (2008). Visualization of Transient Ultra-Weak Protein Self-Association in Solution Using Paramagnetic Relaxation Enhancement. J. Am. Chem. Soc..

[15] Wu K-P, Baum J (2010). Detection of transient interchain interactions in the intrinsically disordered protein α-synuclein by NMR paramagnetic relaxation enhancementw. J. Am. Chem. Soc..

[16] Karamanos TK, Kalverda AP, Thompson GS, Radford SE (2014). Visualization of transient protein-protein interactions that promote or inhibit amyloid assembly. Mol. Cell.

[17] Klare JP, Steinhoff H-J (2009). Spin labeling EPR. Photosynth. Res..

[18] Berliner LJ, Grunwald J, Hankovszky HO, Hideg K (1982). A novel reversible thiol-specific spin label: Papain active site labeling and inhibition. Anal. Biochem..

[19] Gillespie JR, Shortle D (1997). Characterization of long-range structure in the denatured state of staphylococcal nuclease. I. Paramagnetic relaxation enhancement by nitroxide spin labels. J. Mol. Biol..

[20] Iwahara J, Tang C, Marius Clore G (2007). Practical aspects of 1H transverse paramagnetic relaxation enhancement measurements on macromolecules. J. Magn. Reson..

[21] Donaldson LW (2001). Structural characterization of proteins with an attached ATCUN motif by paramagnetic relaxation enhancement NMR spectroscopy. J. Am. Chem. Soc..

[22] Artymiuk P, Blake C (1981). Refinement of human lysozyme at 1.5 Å resolution analysis of non-bonded and hydrogen-bond interactions. J. Mol. Biol..

[23] Mildvan A, Cohn M (1970). Aspects of enzyme mechanisms studies by nuclear spin relaxation induced by paramagnetic probes. Adv. Enzymol. Relat. Areas Mol. Biol..

[24] Wien RW, Morrisett JD, McConnell HM (1972). Spin-label-induced nuclear relaxation. Distances between bound saccharides, histidine-15, and tryptophan-123 on lysozyme in solution. Biochemistry.

[25] Ahn M (2012). Analysis of the native structure, stability and aggregation of biotinylated human lysozyme. PLoS One.

[26] Hagan CL (2010). A non-natural variant of human lysozyme (I59T) mimics the *in vitro* behaviour of the I56T variant that is responsible for a form of familial amyloidosis. Prot. Eng. Design Select..

[27] Xue Y (2009). Paramagnetic relaxation enhancements in unfolded proteins: Theory and application to drkN SH3 domain. Protein Sci..

[28] Schwalbe H (1997). Structural and dynamical properties of a denatured protein. Heteronuclear 3D NMR experiments and theoretical simulations of lysozyme in 8 M urea. Biochemistry.

[29] Klein-Seetharaman J (2002). Long-range interactions within a nonnative protein. Science.

[30] Schmidt MJ (2015). EPR distance measurements in native proteins with genetically encoded spin labels. ACS Chem. Biol..

[31] Baldauf C, Schulze K, Lueders P, Bordignon E, Tampé R (2013). In‐situ spin labeling of His‐tagged proteins: distance measurements under in‐cell conditions. Chem. Eur. J..

[32] Schmidt MJ, Borbas J, Drescher M, Summerer D (2014). A genetically encoded spin label for electron paramagnetic resonance distance measurements. J. Am. Chem. Soc..

[33] Kumita JR (2007). The extracellular chaperone clusterin potently inhibits human lysozyme amyloid formation by interacting with prefibrillar species. J. Mol. Biol..

[34] Brown EM, Pfeffer PE, Kumosinski TF, Greenberg R (1988). Accessibility and mobility of lysine residues in β-Lactoglobulin. Biochemistry.

[35] Andersson LK, Caspersson M, Baltzer L (2002). Control of lysine reactivity in four-helix bundle proteins by site-selective pKa depression: expanding the versatility of proteins by postsynthetic functionalisation. Chem. Eur. J..

[36] Spencer A (1999). Expression, purification, and characterization of the recombinant calcium-binding equine lysozyme secreted by the filamentous fungus *Aspergillus niger*: Comparisons with the production of hen and human lysozymes. Prot. Express. Purif..

[37] Johnson RJ (2005). Rationalising lysozyme amyloidosis: Insights from the structure and solution dynamics of T70N lysozyme. J. Mol. Biol..

[38] Ahn M (2016). The significance of the location of mutations for the native-state dynamics of human lysozyme. Biophys. J..

[39] Sattler M, Schleucher J, Griesinger C (1999). Heteronuclear multidimensional NMR experiments for the structure determination of proteins in solution employing pulsed field gradients. Prog. Nucl. Magn. Reson. Spectrosc..

[40] Delaglio F (1995). NMRPipe: a multidimensional spectral processing system based on UNIX pipes. J. Biomol. NMR.

[41] Sparky 3, University of California, San Francisco (2008).

[42] Function and Data Analysis, Hansen, D. F., http://www.biochem.ucl.ac.uk/hansen/fuda.

