# Supplementary material for: Application of Lysine-specific Labeling to Detect Transient Interactions Present During Human Lysozyme Amyloid Fibril Formation
Source: Sci Rep. 2017 Nov 3;7:15018. doi: 10.1038/s41598-017-14739-5 (PMC5670245; doi:10.1038/s41598-017-14739-5)
Supplement: Supplementary file 1 — Supplementary Information [file 41598_2017_14739_MOESM1_ESM.pdf]

Supporting Information

Application of Lysine-specific Labeling to Detect Transient Interactions Present During Human Lysozyme Amyloid Fibril Formation

Minkoo Ahn[1], Christopher A. Waudby[2], Ana Bernardo-Gancedo[1], Erwin De Genst[1], Anne Dhulesia[1], Xavier Salvatella[3], John Christodoulou[2], Christopher M. Dobson*[1] and Janet R. Kumita*[1]

1 Department of Chemistry, University of Cambridge, Lensfield Road, Cambridge CB2 1EW (UK)
2 Institute of Structural and Molecular Biology, University College London and Birkbeck College, Gower Street, London WC1E 6BT (UK)
3 ICREA and Institute for Research in Biomedicine (IRB Barcelona), The Barcelona Institute of Science and Technology, Baldiri Reixac 10, 08028 Barcelona (Spain)

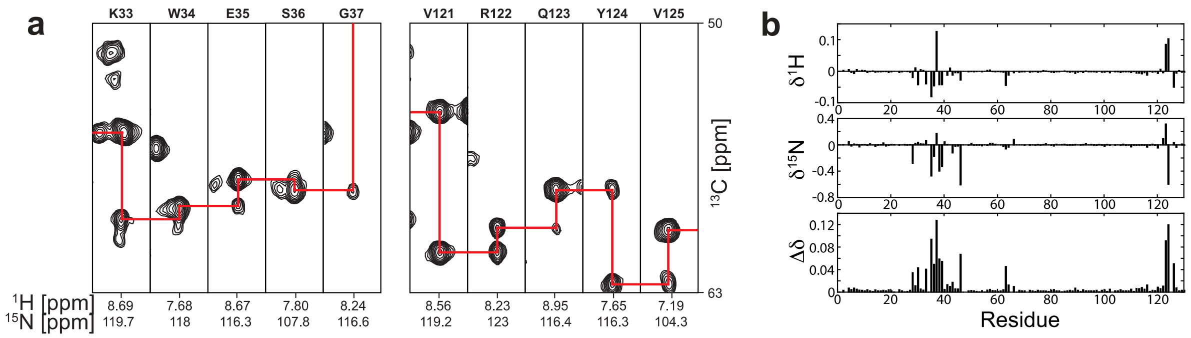

Supplementary Figure S1. Chemical shift assignment and perturbation of SpinHuL. (a) HNCA strip plots of SpinHuL in the vicinity of the residues E35 and Q123. Red horizontal and vertical lines connect the intra-residual and sequential HNCA connectivities and outline the pathway for the sequential assignments. (b) Chemical shift perturbation of SpinHuL from WT human lysozyme. δ^{1}H (top), δ^{15}N (middle) and weighted difference ($\Delta\delta$, bottom) are shown against residue numbers. The relative gyromagnetic ratio of ^{15}N and ^{1}H was used as the weighting factor ($\omega = \gamma_{15N}/\gamma_{1H}$) for calculating $\Delta\delta = ((\delta^{1}H)^2 + (\omega\delta^{15}N)^2)^{0.5}$.

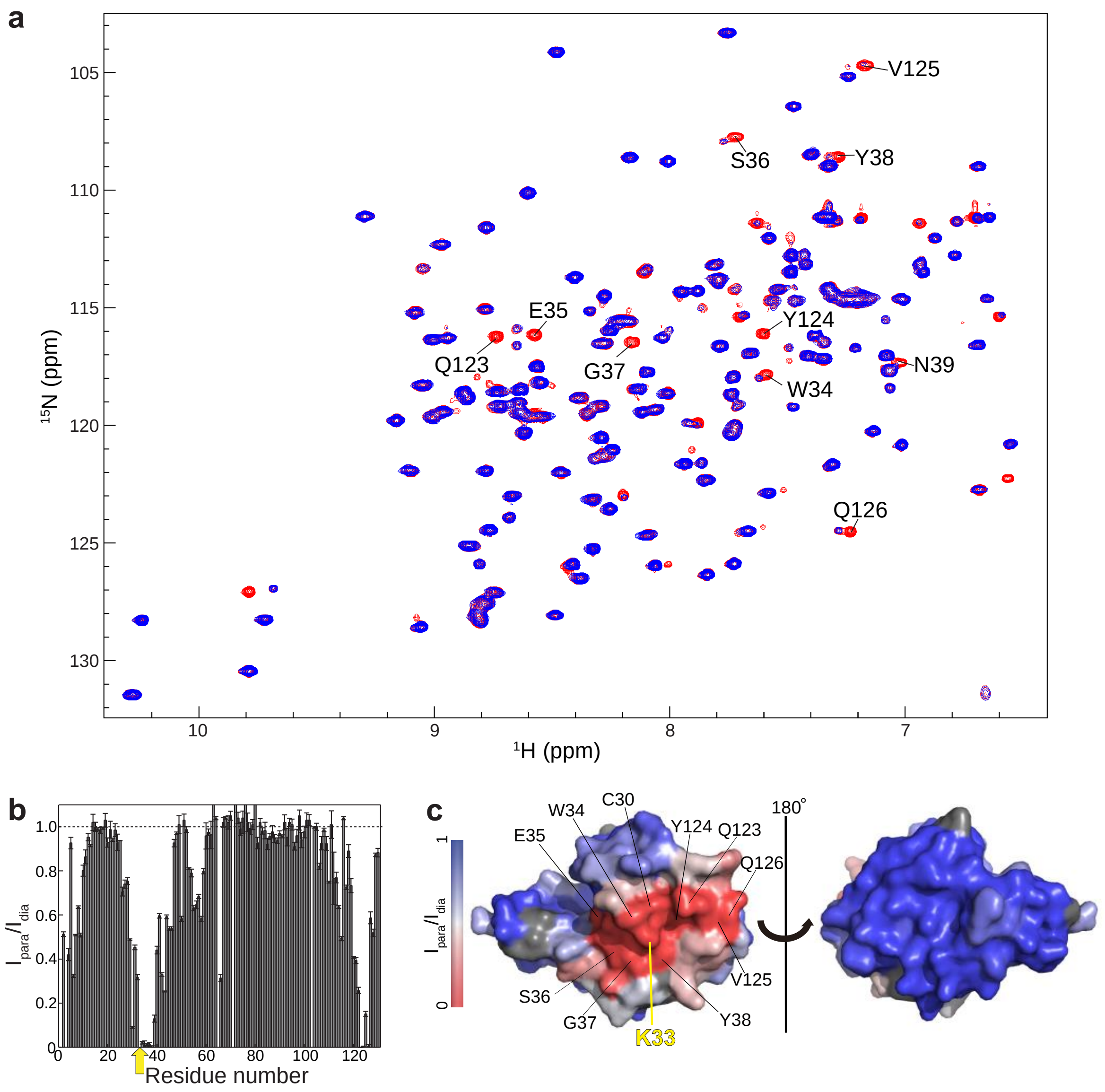

Supplementary Figure S2. Intramolecular PREs for WT lysozyme at pH 5. (a) Overlaid HSQC spectra of paramagnetic (blue) and diamagnetic (red) samples recorded at pH 5.0, 37 °C and 500 MHz. The peaks with significant PRE effects are labeled with their residue numbers. (b) Intensity ratios of the correlation peaks in (a). (c) Structure of spin labeled WT human lysozyme (SpinHuL) colored by the intensity ratios from (b). Experimental values of the intensity ratios higher than 1 are regarded as 1 for coloring. Blue and red colors represent low and high PRE effects, respectively. Residues without experimental PRE values are shown in dark grey. The yellow arrow and line in (b) and (c) show the location of the nitroxide spin label at K33.

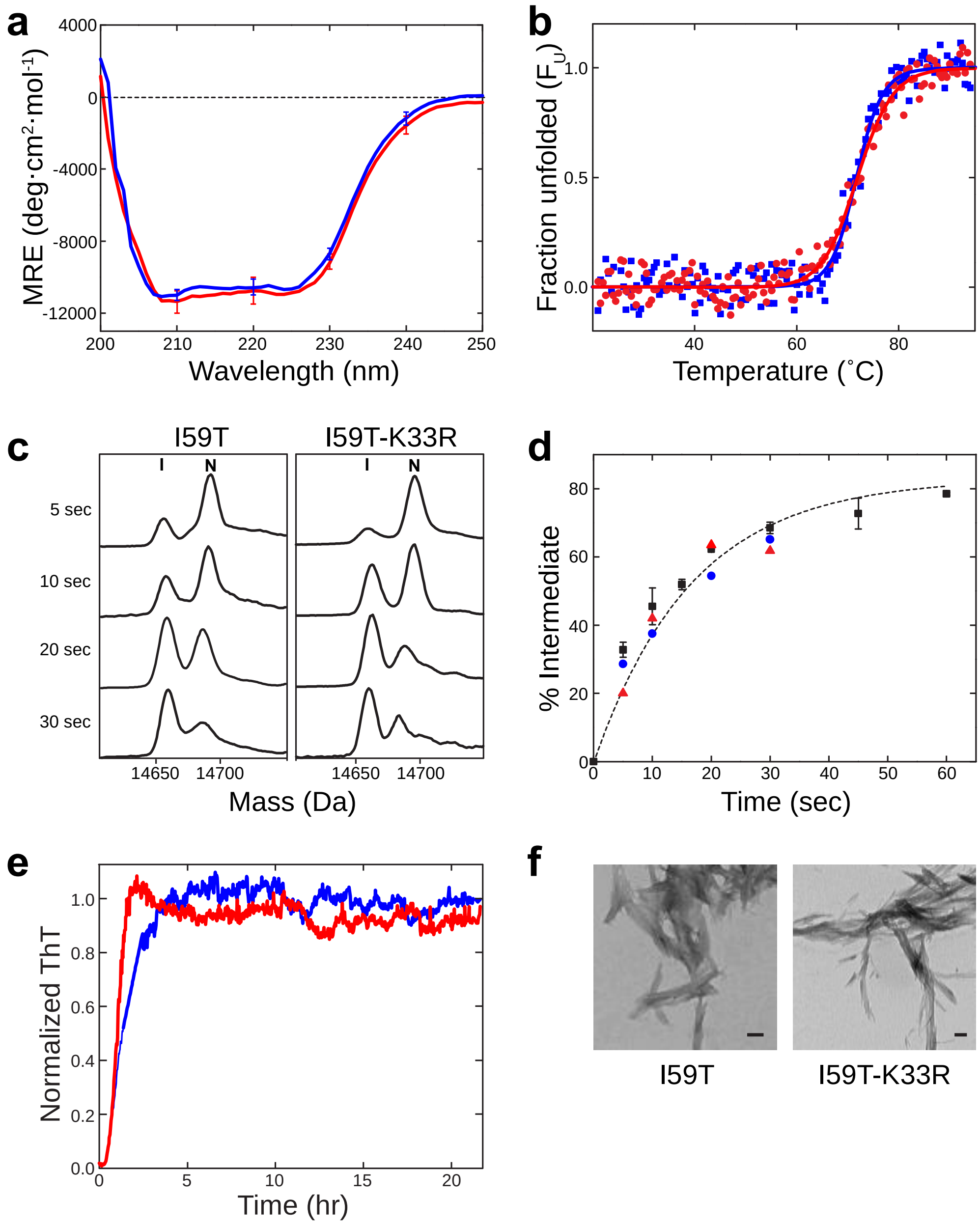

Supplementary Figure S3. Biophysical characterization of the I59T-K33R variant. (a)-(b) Native structure and thermal stability of the I59T and I59T-K33R variants. (a) Secondary structure of I59T (blue) and I59T-K33R (red) recorded by far-UV CD at pH 5.0, 25 °C. The spectra are an average of three independent data sets. (b) Thermal denaturation curves of I59T (blue) and I59T-K33R (red) monitored by far-UV (at 222 nm). Solid lines represent the best fit of the data to a two-state unfolding model. Melting temperature (T_m) values of the two variants from CD thermal denaturation and ANS binding fluorescence are shown in Table S1. The two variants display effectively identical native secondary structure and thermal stability. (c)-(d) Detection of the transient intermediate in the I59T and I59T-K33R variants. (c) HDX monitored by mass spectrometry for I59T and I59T-K33R at 47 °C at different time points. Initially for both variants, a prominent peak corresponding to the deuterated native protein (higher mass species, N) and as time progresses, the intensity of the lower mass species (I) increases. (d) The population of the intermediate state versus time. I59T (blue circles) and I59T-K33R (red triangles) from (c) are shown with the previously published data from I59T (black squares)[1]. I59T-K33R populates the intermediate species in the same way as the I59T variant. (e) Aggregation kinetics of I59T (blue) and I59T-K33R (red) monitored by Thioflavin-T binding in 0.1 M citrate buffer (pH 5.0) at 60 °C with constant stirring. (f) TEM images of the end point fibrils from (e) The black scale bars represent 100 nm. The two variants show essentially identical aggregation propensities to form amyloid fibrils.

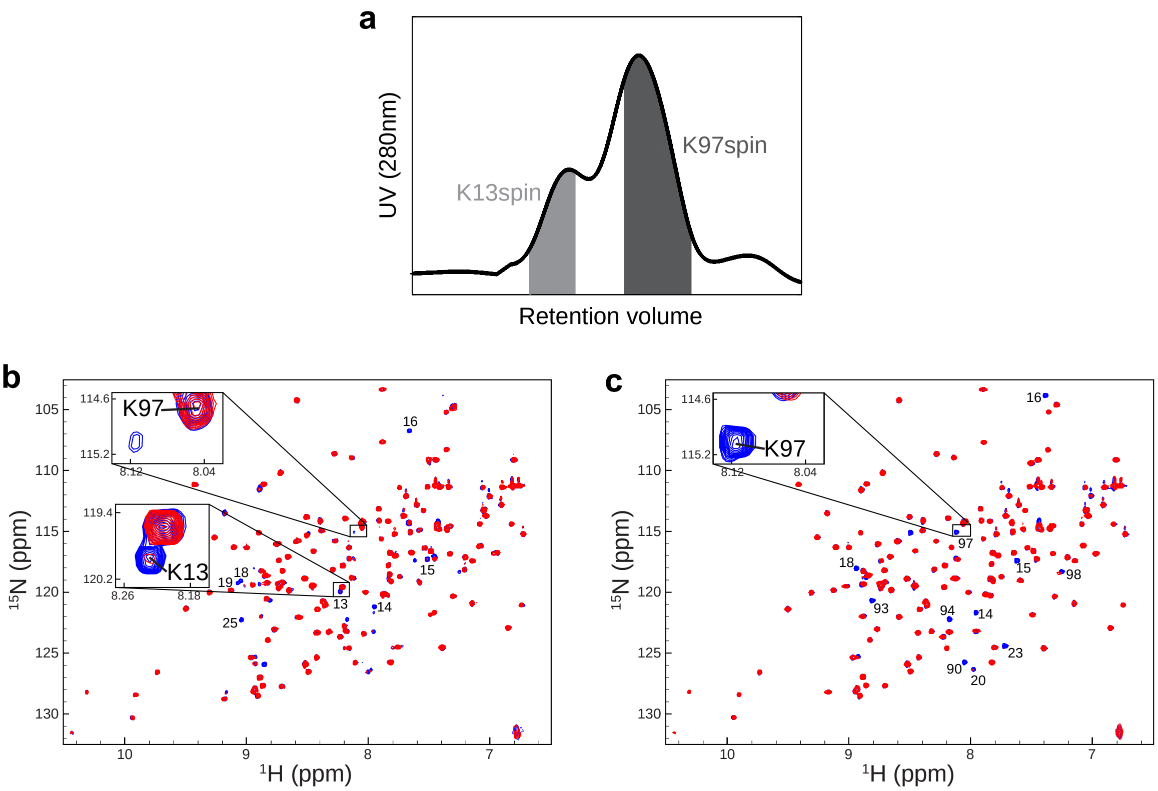

Supplementary Figure S4. Purification and characterization of the I59T-K33R variant with spin labels at K13 (K13spin) and K97 (K97spin). (a) Elution profile from MonoS cation exchange chromatography. Regions of the peaks collected for each sample (to avoid the overlapping region) are indicated for K13spin (light grey) and K97spin (dark grey). (b)-(c) HSQC spectra of paramagnetic (red) and diamagnetic samples (blue) of K13spin (b) and K97spin (c) from (a). The residues that show noticeable intramolecular PRE effects are labeled with assignments. The cross peak of residue 97 shows no PRE effect in the K13spin sample (b) (i.e. no difference in intensities of paramagnetic and diamagnetic samples), whereas there is a strong PRE effects in the K97spin sample (c). The population of the K97spin in the K13spin sample and K13spin in the K97spin sample were less than 10 % and 5 %, respectively.

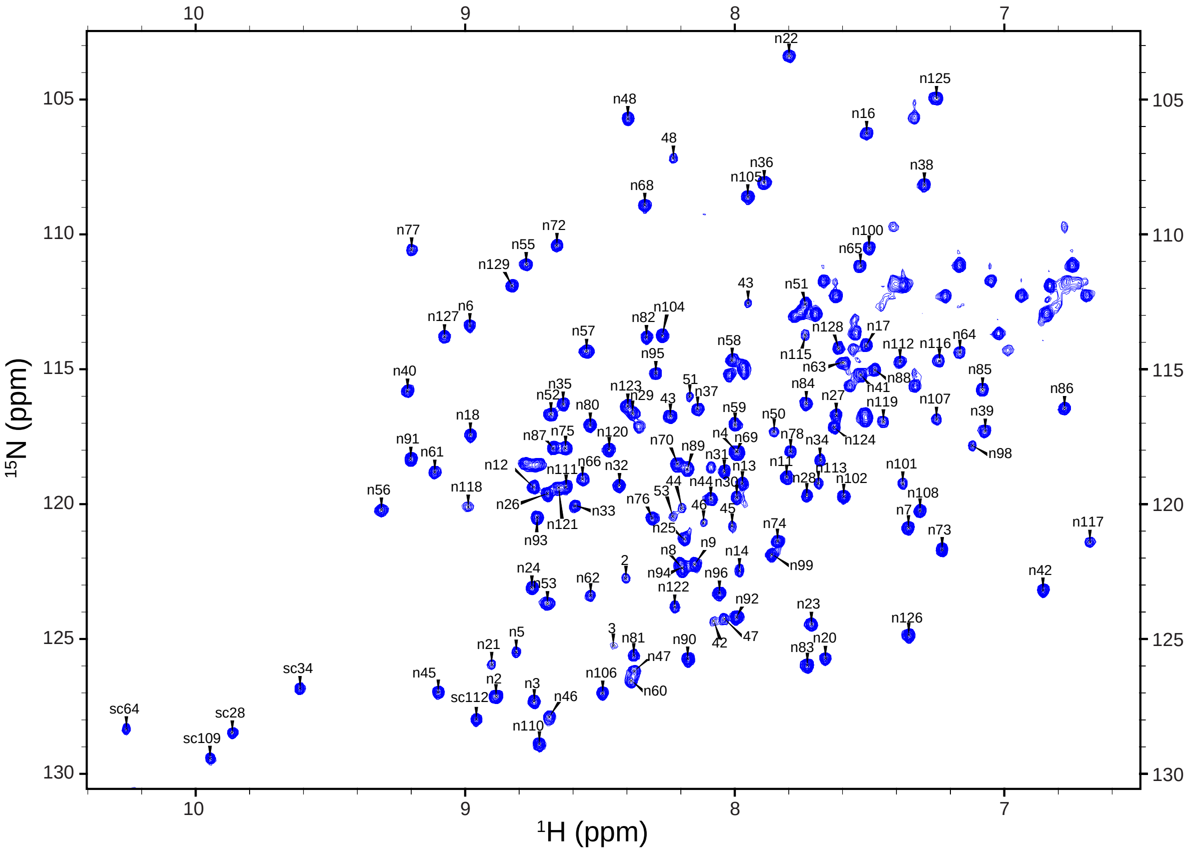

Supplementary Figure S5. Enlarged HSQC spectrum of the I59T at pH 1.2 and 25 °C with assignments. The native peaks and the side-chains are labeled as 'n' and 'sc' with each residue number, respectively, whereas the peaks from the denatured state are only labelled with the residue number.

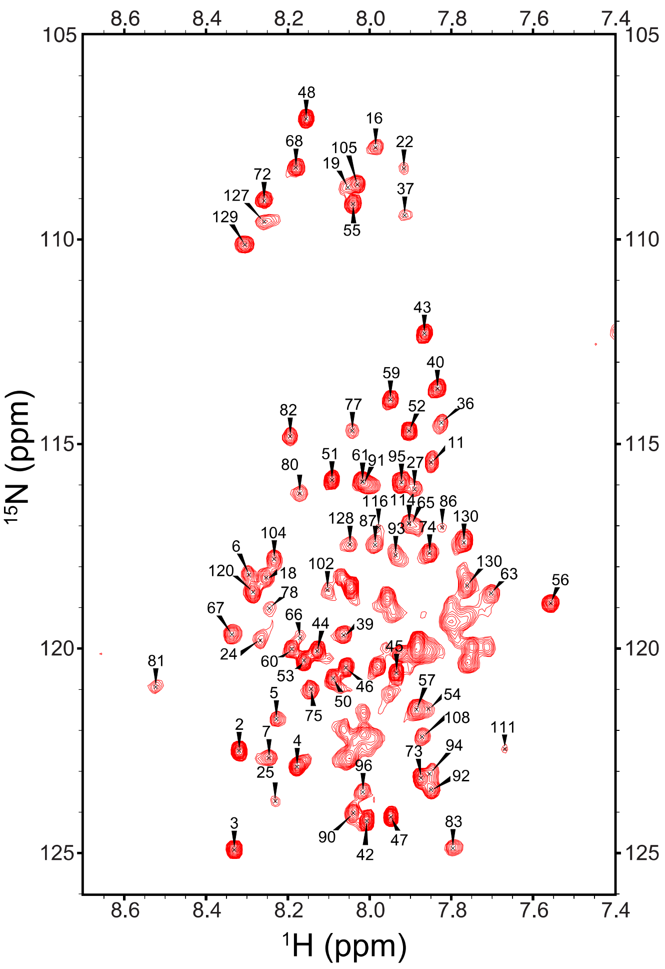

Supplementary Figure S6. Enlarged HSQC spectrum of the I59T at pH 1.2 and 45 °C with assignments. Peaks from the denatured state are shown with the residue number.

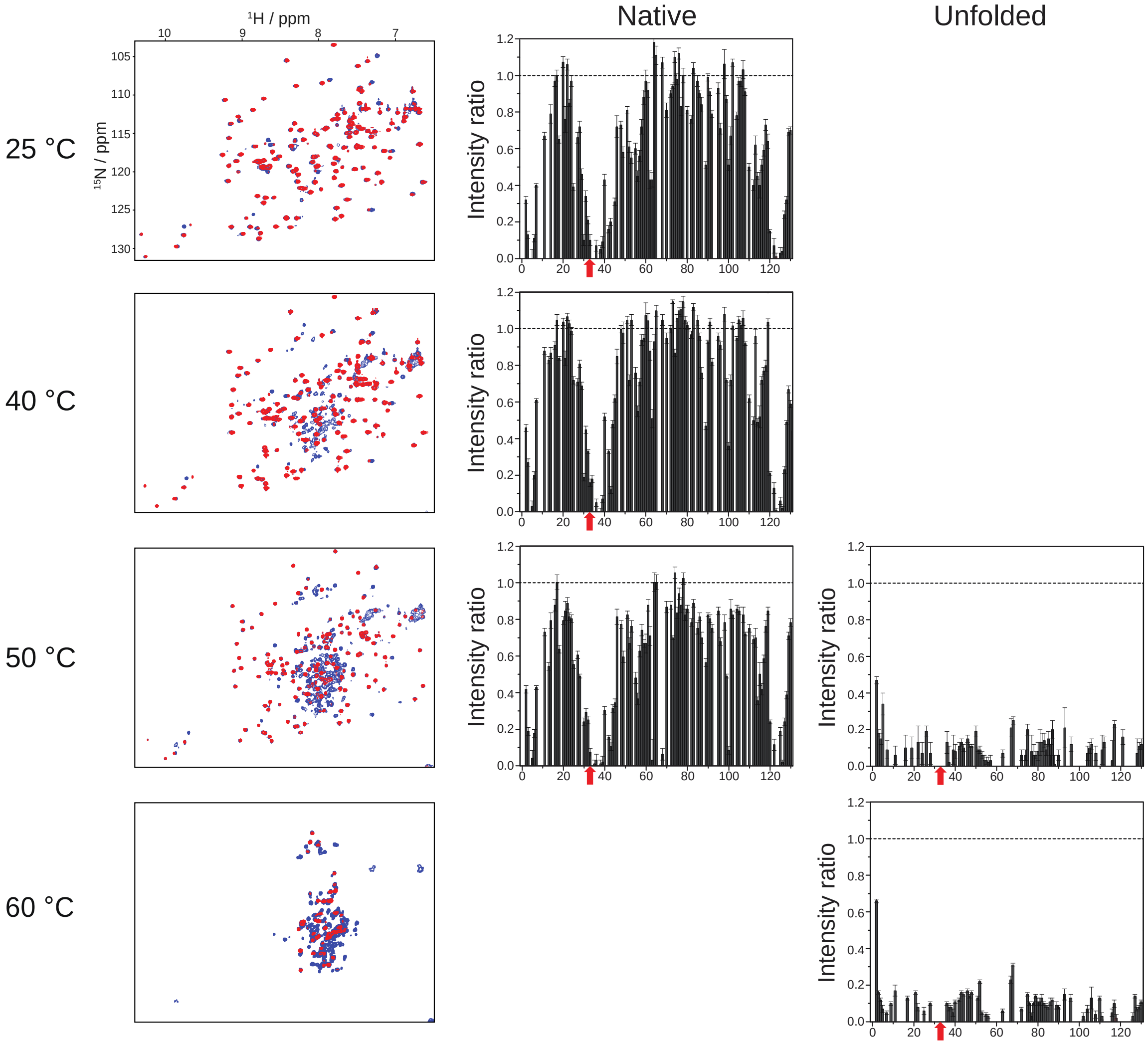

Supplementary Figure S7. Intra-molecular PREs of WT lysozyme at pH 1.2. Overlaid HSQC spectra at the temperatures shown were recorded at 700 MHz. Red and blue peaks indicate the cross peaks from paramagnetic and diamagnetic samples, respectively. Intensity ratios of the cross peaks of the paramagnetic and diamagnetic samples are calculated for native and unfolded peaks at different temperatures. The red arrows represent the location of the nitroxide spin label at K33.

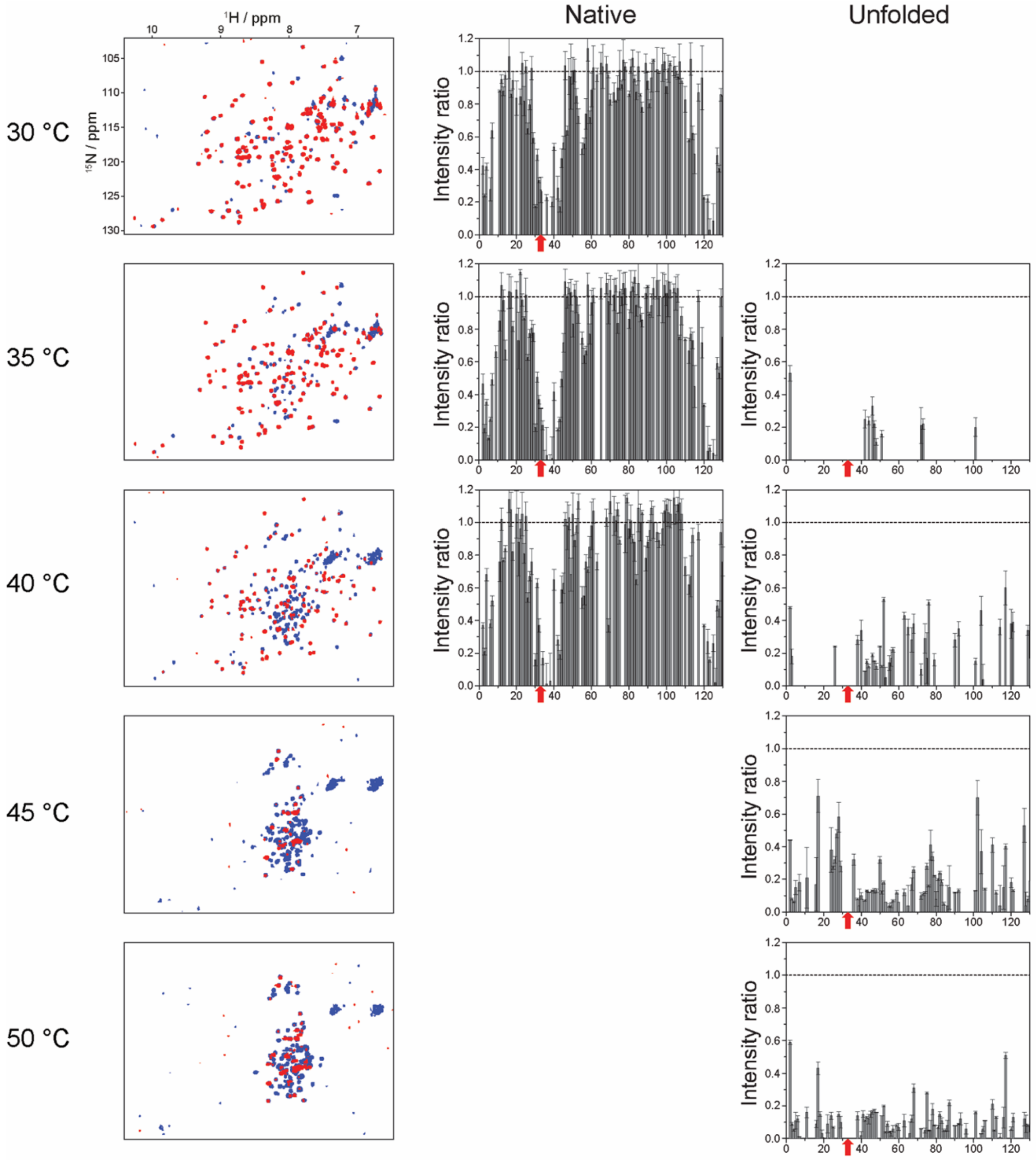

Supplementary Figure S8. Intra-molecular PREs of I59T lysozyme at pH 1.2. Overlaid HSQC spectra at the temperatures shown were recorded at 700 MHz. Red and blue peaks indicate the cross peaks from paramagnetic and diamagnetic samples, respectively. Intensity ratios of the cross peaks of the paramagnetic and diamagnetic samples are calculated for native and unfolded peaks at different temperatures. The red arrows represent the location of the nitroxide spin label at K33.

	$T_{m, Near-UV CD}$	$T_{m, Far-UV CD}$	$T_{m, ANS}$
I59T	71.5 ± 0.4	71.2 ± 0.5	72.5 ± 1.0
I59T-K33R	70.9 ± 0.6	70.6 ± 0.5	71.1 ± 1.0

Supplementary Table S1. Native-state stability of the I59T and I59T-K33R variants determined by thermal denaturation monitored by far- and near-UV CD and ANS binding fluorescence at pH 5.0. All values are in °C.

References

1. Ahn M, *et al.* The Significance of the Location of Mutations for the Native-State Dynamics of Human Lysozyme. *Biophys J* **111**, 2358-2367 (2016).
